# Effect of the prism and Maddox rod test as the surgical target for type III acute acquired comitant esotropia

**DOI:** 10.3389/fmed.2024.1389201

**Published:** 2024-04-15

**Authors:** Huihang Wang, Weidong Zheng

**Affiliations:** ^1^Department of Ophthalmology, The First Affiliated Hospital, Fujian Medical University, Fuzhou, China; ^2^Department of Ophthalmology, National Regional Medical Center, Binhai Campus of the First Affiliated Hospital, Fujian Medical University, Fuzhou, China

**Keywords:** acute acquired concomitant esotropia, prism and Maddox rod test, examination method, prism and alternate cover test, deviation angle

## Abstract

**Introduction:**

This study aims to explore more accurate and efficient examination methods to provide precise target surgical measurements for patients with type III acute acquired comitant esotropia (AACE).

**Methods:**

The study conducted a retrospective analysis of 108 patients diagnosed with AACE who received surgical treatment at the Department of Ophthalmology, the First Affiliated Hospital of Fujian Medical University, from January 2018 to September 2023. All patients underwent examinations of the deviation angle, including the Hirschberg test, prism and Maddox rod test (PMT), and prism and alternate cover test (PACT). For the PACT, the minimum value (PACTmin) and maximum value (PACTmax) were obtained based on differences in examination methods, as well as the deviation angle range (PACT range), which represents the difference between PACTmax and PACTmin. Postoperatively, these patients were followed up for at least 6 months to assess changes in eye position and whether diplopia symptoms recurred.

**Results:**

In both near and distant examinations, the results of PACTmax were significantly greater than those of PACTmin (*p* < 0.001), while the deviation angles obtained from PACTmax and PMT showed no significant statistical difference [*p* = 0.689 (33 cm), *p* = 0.436 (5 m)]. There was a strong linear correlation between PACTmin and PMT at both near (*R* = 0.8887) and distant (*R* = 0.8950) distances, but each PACTmin corresponded to multiple PMT values. There was no significant difference between the results of PACT range at near and distant distances (*p* = 0.531). The deviation angles obtained by PMT and PACTmin significantly decreased postoperatively compared to preoperative values, and diplopia disappeared in all patients, with alternative cover test showing no movement or presenting as an esophoria state.

**Conclusion:**

The PMT can provide precise target surgical measurements for type III AACE, making it a fast, effective, and cost-efficient examination method. It is worthy of being promoted and applied in clinical practice.

## 1 Introduction

Acute acquired comitant esotropia (AACE) is a specific type of esotropia commonly seen in older children or adults with mature visual development, often accompanied by symptoms such as ipsilateral diplopia, headache, and eye fatigue (1–3). Traditionally, AACE is classified into three types (4): Type I, Swan type; Type II, Burian-Franceschetti type; and Type III, Bielschowsky type. In a retrospective study based on 48 children, that took place from 2000 to 2013, Buck et al. reclassified AACE into 7 types (5), however, this classification is not yet widely recognized. Therefore, this study still uses the traditional classification.

Swan type, first proposed by Swan in 1947 (4), is characterized by AACE that occurs following the interruption of binocular single vision, such as after treatment for amblyopia, occlusion after unilateral corneal injury or surgery, or resolution of eyelid swelling after ocular trauma. Burian-Franceschetti (4) type presents with sudden onset of large-angle esotropia without apparent triggers, no accommodative factors, and no significant refractive error or mild hyperopia. It often occurs in situations of physical or psychological stress. Bielschowsky type (4, 6), first reported by Bielschowsky in 1922, occurs in adults or older children with uncorrected myopia greater than −5.00D, but later studies have found that AACE type III also occurs in people with low and intermediate myopia (7, 8). It results from imbalanced convergence and divergence due to excessive near work, leading to accommodative esotropia. Early manifestations include esotropia with ipsilateral diplopia when looking at distance with fusion but without diplopia when looking at near. As the condition progresses, diplopia may gradually appear even at near. With the proliferation of electronic devices and the prolonged period of working and studying from home due to the COVID-19 pandemic, the incidence of AACE has increased (9, 10). In recent years, the majority of AACE patients presenting for treatment have been Type III (11, 12).

The primary treatment goals for AACE patients are to eliminate diplopia and correct esotropia. Currently, the main treatment methods for AACE include prism correction, botulinum toxin type A injection into the extraocular muscles, and surgical treatment. Prism correction and botulinum toxin injection are often used in the early stages of the disease when diplopia is not severe and the degree of esotropia is small or unstable. For most patients with stable esotropia, surgery is the main treatment method, but there is a risk of postoperative undercorrection, recurrence, and the need for multiple surgeries (13). Regarding the phenomenon of postoperative undercorrection or recurrence, some scholars believe it is due to the characteristic of AACE patients “eating up prisms,” making it difficult to obtain accurate target surgical measurements during examinations (14). Therefore, some scholars suggest increasing the surgical increment for AACE patients to reduce the possibility of postoperative recurrence or undercorrection (15, 16). Additionally, some researchers have found that prism adaptation tests can reveal larger degrees of deviation in AACE patients (13), but these tests are time-consuming, expensive, and currently lack consensus in clinical practice. For AACE patients, we believe that determining precise target surgical measurements preoperatively is crucial for surgical success.

Currently, the commonly used clinical methods for assessing strabismus include the Hirschberg test, prism and alternative cover test (PACT), prism and Maddox rod test (PMT), and prism adaptation test (PAT) (14, 17–19). However, there are few published research papers that use the measurement results of PMT as the target surgical amount for type III AACE. Therefore, this study focuses on type III AACE, which is the most common type, using the measurement results of PMT as the target surgical measurements for surgical treatment, while observing postoperative efficacy. The study aims to explore suitable examination methods and treatment modalities for type III AACE.

## 2 Materials and methods

### 2.1 Subjects

This study retrospectively analyzed 108 cases of type III AACE patients who underwent surgical treatment at the Department of Ophthalmology, the First Affiliated Hospital of Fujian Medical University, from January 2018 to September 2023. The study adhered to the Helsinki Declaration and was approved by the Medical Ethics Committee of the First Affiliated Hospital of Fujian Medical University. Patients or their guardians were informed and consented, and signed informed consent forms. Type III AACE was defined as acute non-accommodative esotropia occurring in older children and adult patients. Early manifestations included diplopia at distance but not at near, with the degree of esotropia gradually increasing as the disease progressed, resulting in diplopia at both distance and near. The deviation angle was the same in all gaze directions, and there were no limitations in ocular motility.

### 2.2 Examination

This study retrospectively reviewed data from 108 cases of type III AACE patients, collecting information including patient gender, age, duration of illness, refractive error, and deviation angle measured using various methods. At the initial visit, all patients underwent cycloplegic refraction using 0.5% tropicamide eye drops to obtain refractive values. The refractive values were converted to spherical equivalent (SE), calculated as the algebraic sum of the sphere and half of the cylinder. Refractive correction was performed using the maximum positive diopter lens that provided the best-corrected visual acuity (BCVA) according to the Maximum Plus to Maximum Visual Acuity (MPMVA) principle, with BCVA recorded as LogMAR visual acuity. Ocular anterior segment examination using a slit lamp (Haag-Streit AG, BQ 900) was conducted to exclude organic lesions. Scanning laser ophthalmoscopy (SLO, Heidelberg Engineering, Heidelberg, Germany) was performed for fundus examination to rule out fundus diseases. All patients underwent orbital and cranial imaging examination, and were thoroughly questioned about common causes of type III AACE; patients with orbital diseases, head trauma, or central nervous system abnormalities were excluded from this study. Additionally, patients with residual or secondary esotropia after strabismus correction surgery, those with combined extraocular muscle dysfunction, A-V pattern strabismus, monocular suppression, or dissociated vertical deviation were also excluded from this study.

**Figure 1:**
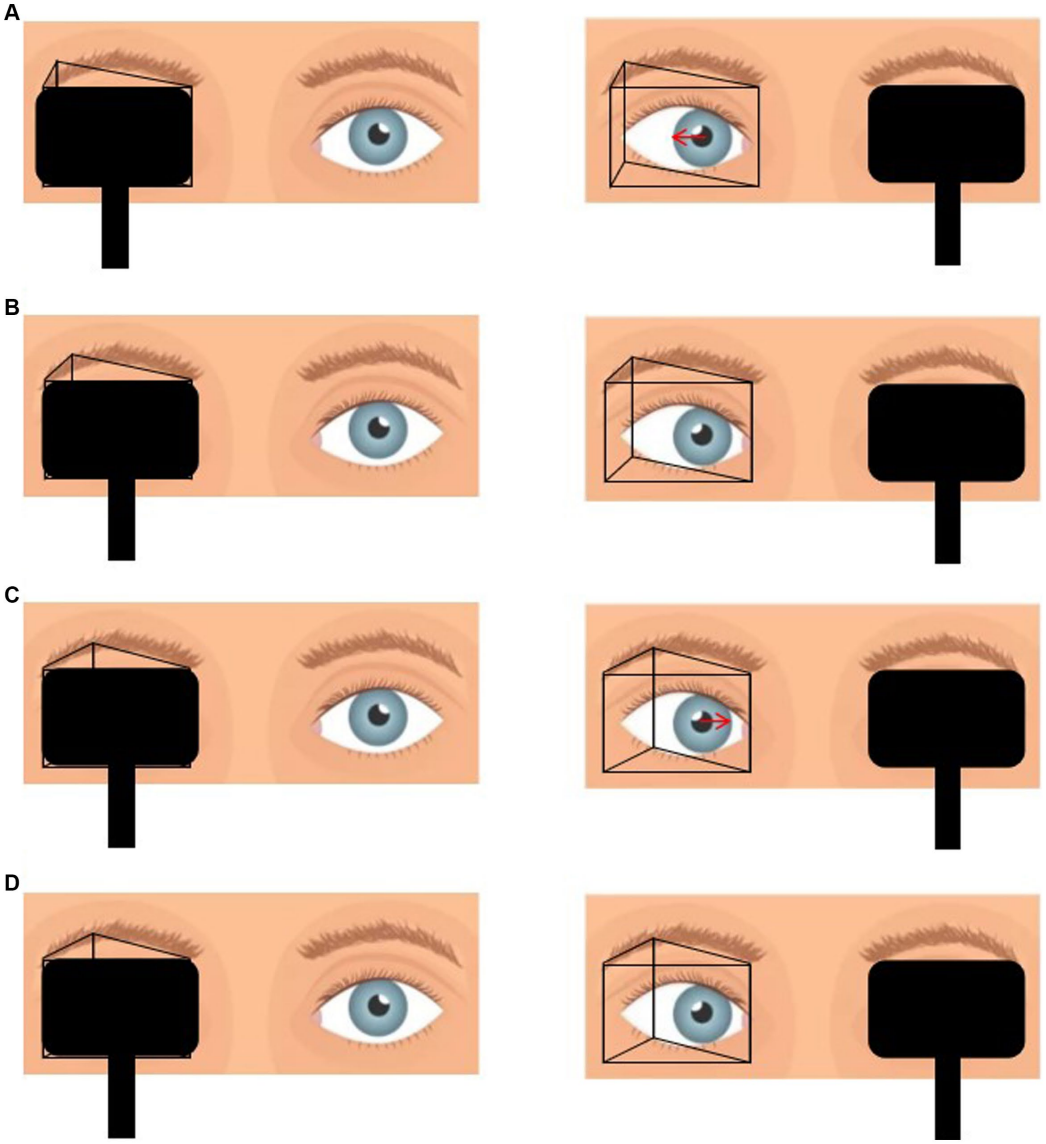
**(A)** Placement of prism in front of the one eye with alternate cover test reveals that the eye position is from inside to center, which suggests that the prismaticity is still insufficient. **(B)** Gradually increase the prismaticity until the eye position immobile during alternate cover test, at which point the prismaticity is recorded as PACTmin. **(C)** Continue to increase the prismaticity until the eye position is from outside to center. **(D)** Decrease the prismaticity until the eye position was immobile, at which point the prismaticity was recorded as PACTmax.

**Figure 2:**
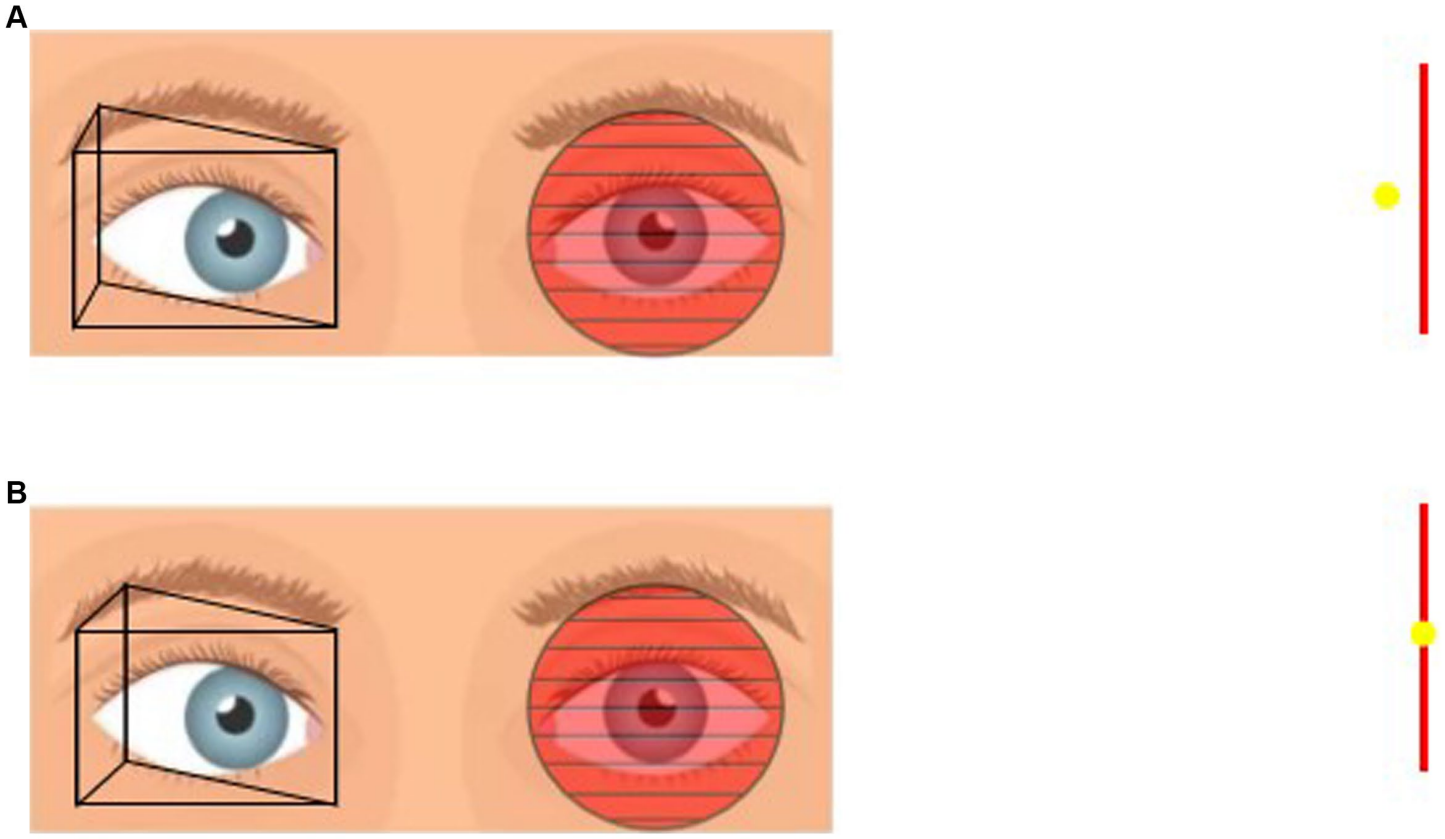
**(A)** Placement of prism in front of the one eye and Maddox rod in front of the other eye. If the patient sees the vertical line on the left and the point light on the right then the prismaticity is still insufficient. **(B)** Gradually increase the prismaticity until the vertical line and the point light fit together, at which point the prismaticity was recorded as PMT.

In this study, all patients underwent deviation angle examination based on refractive correction. We used three methods to assess the deviation angle: the HT, PACT and PMT. The HT was used to assess near deviation by placing a point light source in front of the patient at a distance of 33 cm and determining the angle of ocular deviation based on the position of the corneal light reflex. For the PMT, a red Maddox rod was placed horizontally in front of the patient’s eye, and a base-out prism was placed in front of the other eye. The patient was instructed to fixate on a distant target (5 m) and a near target (33 cm). The base-out prism was gradually increased until the vertical streak overlapped with the point light source, and the PMT values were recorded at 5 m and 33 cm. During the PACT, an accommodative target was set at both near (33 cm) and distant (5 m) distances. The patient was instructed to fixate on the target while alternating eye occlusion, and the base-out prism power was gradually increased. The prism power at which the eye movement disappeared after uncovering and returning to fixation was recorded as PACTmin. Then, the prism power was gradually increased until eye movement reappeared after uncovering and returning to fixation, and the prism power at this point was recorded as PACTmax. The examination process of PMT and PACT is shown in Figures 1, 2. This process was repeated to measure the PACT values when the patient fixated at 15° to the left, 15° to the right, 25° upward, and 25° downward. Preoperatively, HT, PMT, and PACT examinations were performed at three different time points. To expose the maximum deviation angle, the highest value obtained from three consecutive measurements was used for each examination method to eliminate the phenomenon of “prism adaptation” in type III AACE patients.

### 2.3 Surgery and follow-up assessment

All surgeries were performed by the same experienced ophthalmologist (WD. Zheng). We used the results of the PMT examination as the target surgical measurements for AACE patients undergoing surgery. Based on the magnitude of the deviation angle, unilateral medial rectus recession (MRrec) of the non-dominant eye or combined MRrec and lateral rectus resection (LRres) of the non-dominant eye were performed. For patients with PMT results exceeding 80 prism diopters (PD), bilateral MRrec combined with non-dominant eye LRres was considered. The surgical dosage was determined according to the Parks scale. All patients in this study were able to cooperate with surgery under local anesthesia. During surgery, patients needed to wear refractive correction glasses and fixate on targets at 5 m and 33 cm to confirm the disappearance of diplopia. We reserved adjustable sutures and ligated them after observing that the patients had no diplopia in all directions and orthotropia in the distance position, orthotropia or slight exotropia in the near position. No adjustments were made in all patients in this study eventually. Follow-up examinations were conducted at 1 day, 1 week, 1 month, 3 months, and 6 months postoperatively, including evaluations of HT, PMT, and PACT. Patients with a follow-up period exceeding 6 months were also followed up via telephone. Surgical success was defined as the disappearance of diplopia postoperatively, and residual objective and subjective deviation angle were evaluated based on PACT min and the far and near horizontal deviation measured by PMT.

### 2.4 Statistics

The data were analyzed using descriptive statistics, reporting mean and standard deviation for normally distributed data, and counts and percentages for categorical data. In the study of deviation angle examination methods, paired-sample t-tests and two independent-sample t -test were used to analyze differences in near-distance disparity (NDD) measured by different examination methods. Correlation analysis was conducted using linear regression and Pearson correlation coefficient (r). All data analyzes were performed using SPSS (StatLab, SPSS version 25.0) and Prism (StarBio-LLC, Prism 9 version 9.4.1). A two-tailed test was used, with *p* < 0.05 considered statistically significant. The Pearson correlation coefficient (r) values were interpreted as follows: 0 ≤ |r| < 0.3 indicates weak linear correlation, 0.3 ≤ |r| < 0.8 indicates moderate linear correlation, and 0.8 ≤ |r| ≤ 1 indicates strong linear correlation.

## 3 Results

### 3.1 Study population

This study included a total of 108 cases of type III AACE patients, all of whom were Chinese. The basic information and clinical characteristics of the patients are summarized in Table 1. The patients were all older children and adults, with an average age of 26.86 ± 11.59 years (ranging from 10 to 53 years), and the male proportion was 56.48%. The LogMAR BCVA of both eyes of all patients was less than 0.10, with no significant statistical difference in BCVA and SE between the eyes (*p* values were 0.654 and 0.597, respectively), and the proportion of eyes with refractive disparities was only 5.55%. For detailed information, please refer to Table 1.

**Table 1:** Epidemiology and clinical characteristics of the type III AACE patients.

Subjects (*N* = 108)	Values	Mean ± SD	*p*-value
Sex, male No. (%)	61 (56.48%)		
Sex, female No. (%)	47 (43.52%)		
Age (years)	10–53	27.03 ± 10.94	
Duration (months)	1–120	33.96 ± 30.12	
BCVA OD (logMAR)	−0.079–0.097	−0.0050 ± 0.044	0.654
BCVA OS (logMAR)	−0.079–0.097	−0.0068 ± 0.042	
SE OD (D)	−11.5–1.25	−4.23 ± 2.09	0.597
SE OS (D)	−11.25–2	−4.18 ± 2.29	
Dominant eye OD No. (%)	58 (53.70%)		
Dominant eye OS No. (%)	50 (46.30%)		
Anisometropia No. (%)	6 (5.55)		

BCVA, best-corrected visual acuity; OD, right eye; OS, left eye; logMAR, logarithm of the minimum angle of resolution; SE, spherical equivalent; D, diopter; No., number of patients; P, Paired samples t-test; N, number of cases.

### 3.2 Epidemiological characteristics

**Figure 3:**
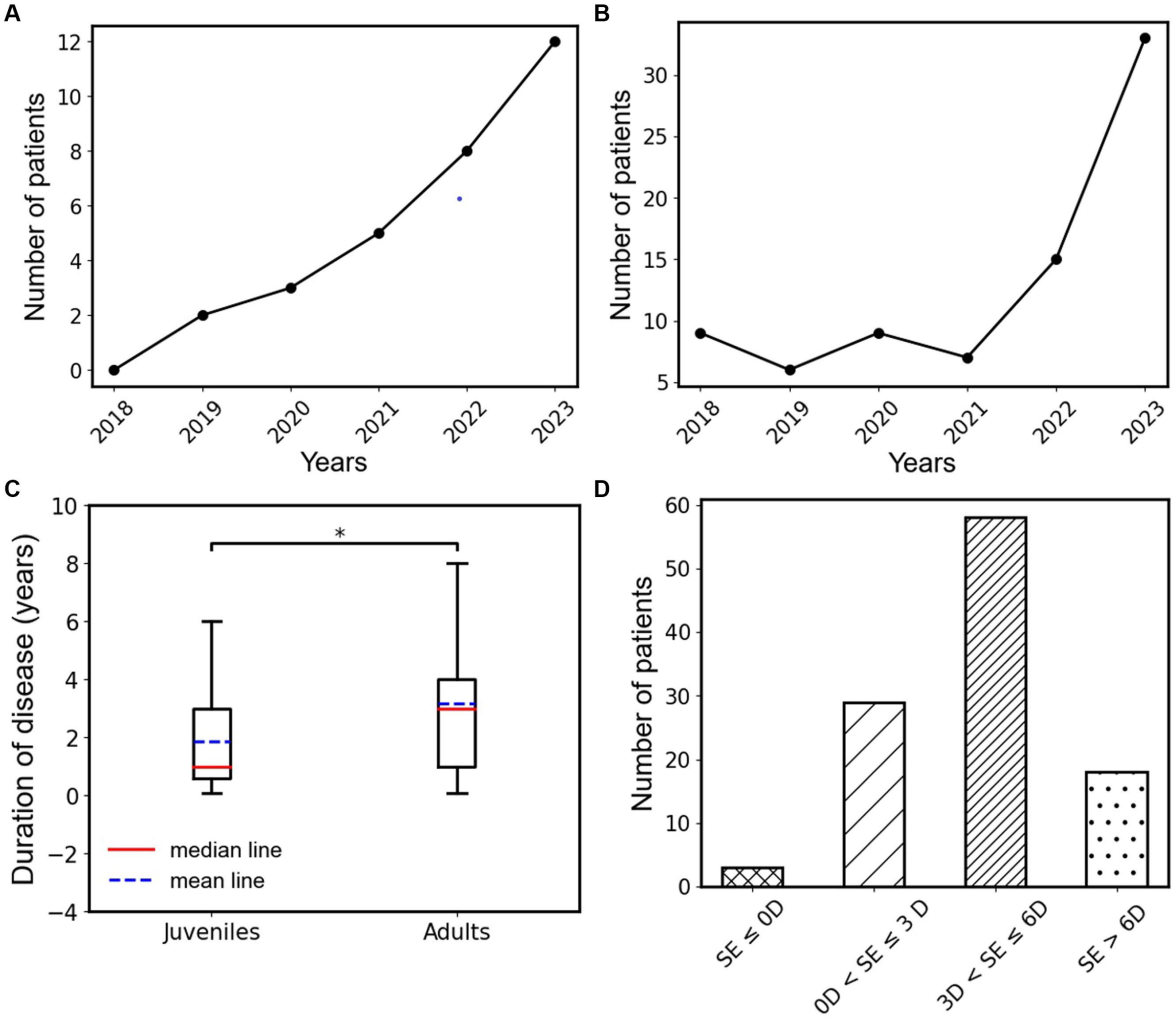
**(A)** Annual change trend in the number of type III AACE patients in the juvenile group from 2018 to 2023. **(B)** Annual change trend in the number of type III AACE patients in the adult group from 2018 to 2023. **(C)** The box plots of disease duration in juvenile and adult groups of type III AACE. **(D)** Binocular mean equivalent spherical distribution of type III AACE patients; *indicates the *p* < 0.05.

This study included patients who underwent treatment from January 2018 to September 2023. Statistical analysis revealed a gradual increase in the number of type III AACE patients treated each year since 2018 (Figure 3). Furthermore, we observed a sudden surge in the number of type III AACE patients after 2020, possibly due to the increased use of close-up activities such as remote work or online teaching resulting from the outbreak of the COVID-19 pandemic. We categorized type III AACE patients into adult and pediatric groups and conducted epidemiological analyzes separately. We found that the number of pediatric patients gradually increased after the COVID-19 pandemic (Figure 3A), while the number of adult patients significantly increased one to two years after the outbreak (Figure 3B). To further explore the differences between the two groups, we statistically analyzed the duration of illness at the time of initial diagnosis and found that the duration of illness in the pediatric group was significantly shorter than that in the adult group, with a statistically significant difference (*p* = 0.015) (Figure 3C). The average spherical equivalent refractive error of both eyes of type III AACE patients was predominantly moderate myopia, followed by mild myopia, with high myopia, hyperopia, and emmetropia being rare. This is consistent with our previous research findings on independent risk factors for type III AACE (14).

### 3.3 Comparison of different examination methods

In this study, we employed various methods of deviation angle examination to measure the degree of ocular deviation in patients with type III AACE, including HT, PACT, and PMT. Among them, HT examined only the ocular deviation angle at near distance, while the other two methods provided measurements of ocular deviation angles at both near and far distances. These results help us better understand the characteristics of type III AACE and select appropriate surgical target amounts. Table 2 visually compares the mean ocular deviation angles at near and far distances obtained from PACT and PMT, along with standard deviations and numerical ranges. As shown in Table 2, there were no significant statistical differences (*p* > 0.05) in the ocular deviation angles measured at near and far distances by all examination methods, including PMT, PACTmin, and PACTmax, indicating that the ocular deviation angles of type III AACE patients are not correlated with distance.

**Table 2:** The results of different inspection distances and methods.

Methods	Near deviation (33 cm, PD)	Distance deviation (5 m, PD)	t	*p*-value
PMT	45.69 ± 24.19 (10 to 115)	46.70 ± 24.13 (12 to 115)	−0.486	0.628
PACTmin	28.65 ± 17.54 (0 to 70)	29.08 ± 17.83 (4 to 75)	−0.282	0.778
PACTmax	44.37 ± 24.30 (8 to 105)	44.19 ± 23.11 (10 to 110)	0.085	0.932

PACT, prism and alternative cover test; PMT, prism and Maddox rod test; PD, prism diopter.

**Figure 4:**
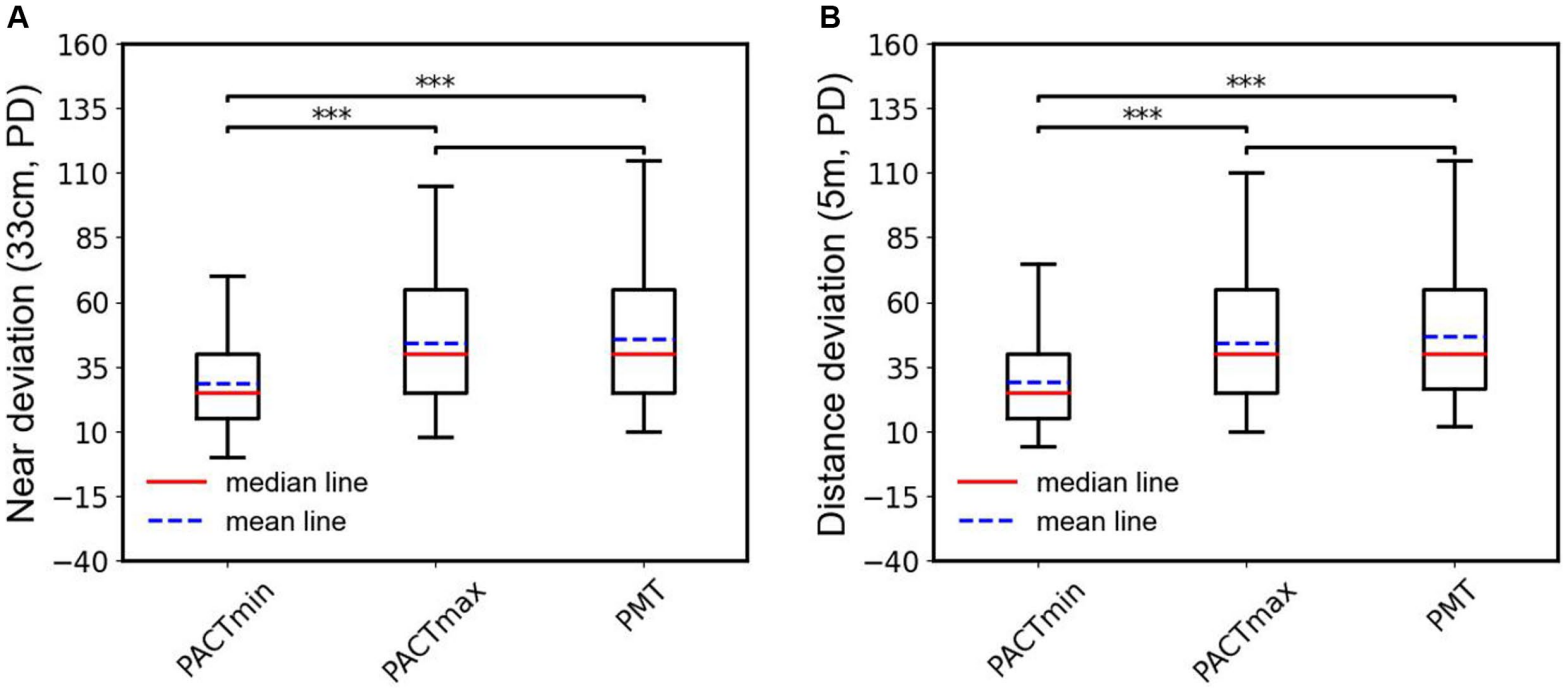
**(A)** Box plots and statistical analysis of the PACTmin, PACTmax and PMT in near range. **(B)** Box plots and statistical analysis of the PACTmin, PACTmax and PMT in distance range; ***indicates the *p* < 0.001.

**Figure 5:**
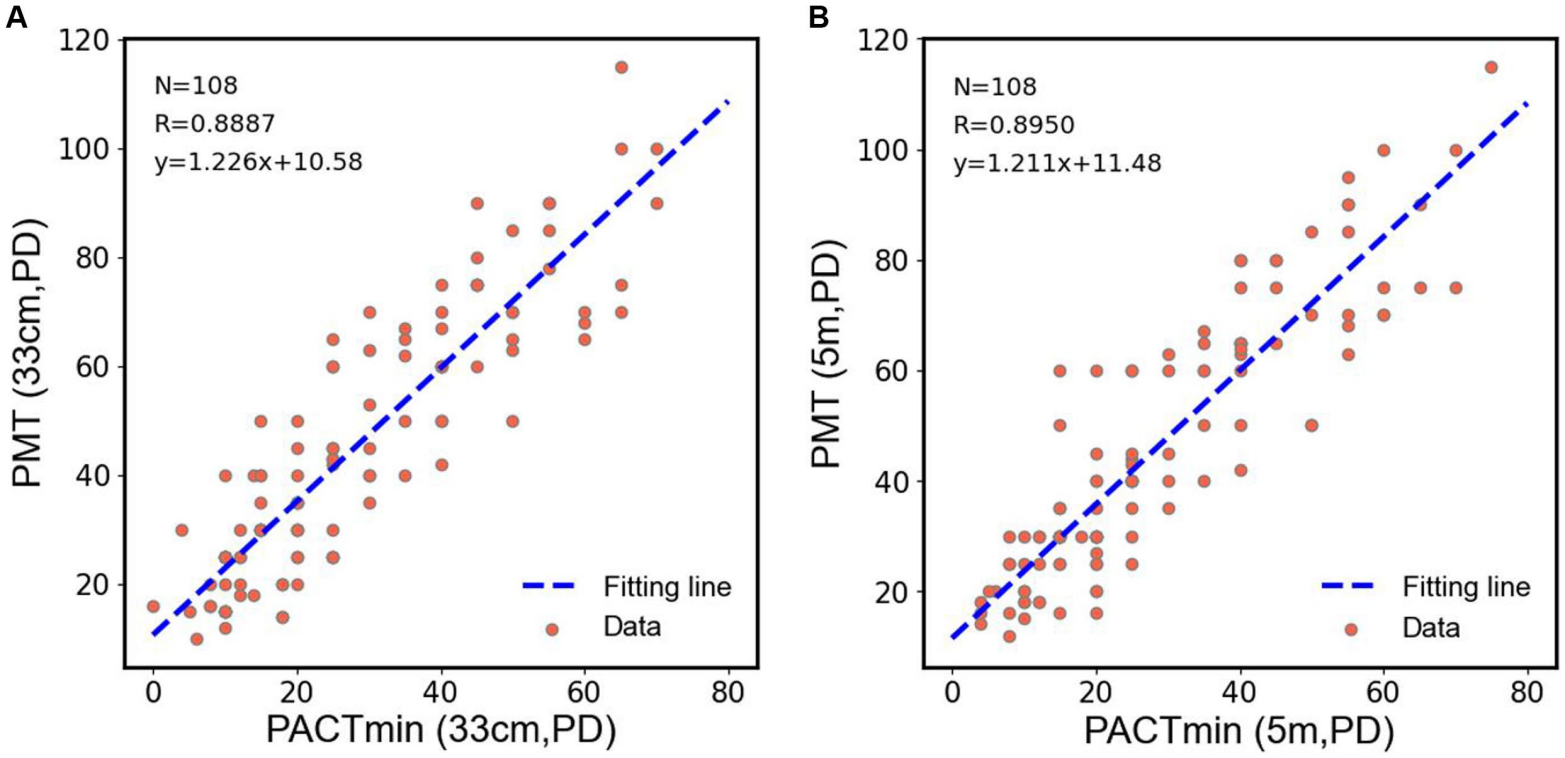
**(A)** Scatter plots were drawn by PACTmin and PMT of each patient in near range, and linear regression and correlation analysis were performed for the scatter plots. **(B)** Scatter plots were drawn by PACTmin and PMT of each patient in distance range, and linear regression and correlation analysis were performed for the scatter plots.

To further observe the differences among various examination methods in patients with type III acute acquired comitant esotropia (AACE), we conducted additional statistical analyzes. As shown in Figure 4A, in the examination at near distance (33 cm), we performed paired t-tests on PMT, PACTmax, and PACTmin for each patient. The results showed that PACTmax was significantly greater than PACTmin, with extremely significant statistical differences (*p* < 0.001); PMT was also significantly greater than PACTmin, with extremely significant statistical differences (*p* < 0.001); however, there was no significant statistical difference between PMT and PACTmax (*p* = 0.689). Similar phenomena were observed in the examination at far distance (5 m), as shown in Figure 4B. PACTmax was significantly greater than PACTmin, with extremely significant statistical differences (*p* < 0.001); PMT was also significantly greater than PACTmin, with extremely significant statistical differences (*p* < 0.001); there was no significant statistical difference between PMT and PACTmax (*p* = 0.436). We further conducted linear regression analysis between PMT and PACTmin, as illustrated in Figure 5. Strong linear correlations were observed between PMT and PACTmin at both near and far distances. According to the linear regression results, at near distance, PMT = 1.226 × PACTmin +10.58, with R = 0.8887. At far distance, PMT = 1.211 × PACTmin +11.48, with R2 = 0.8950. The results indicate that larger PACTmin values correspond to larger PMT values, but there is not a one-to-one correspondence between PACTmin and PMT values. For different patients, although the PACTmin values are the same, they may correspond to different PMT values.

**Figure 6:**
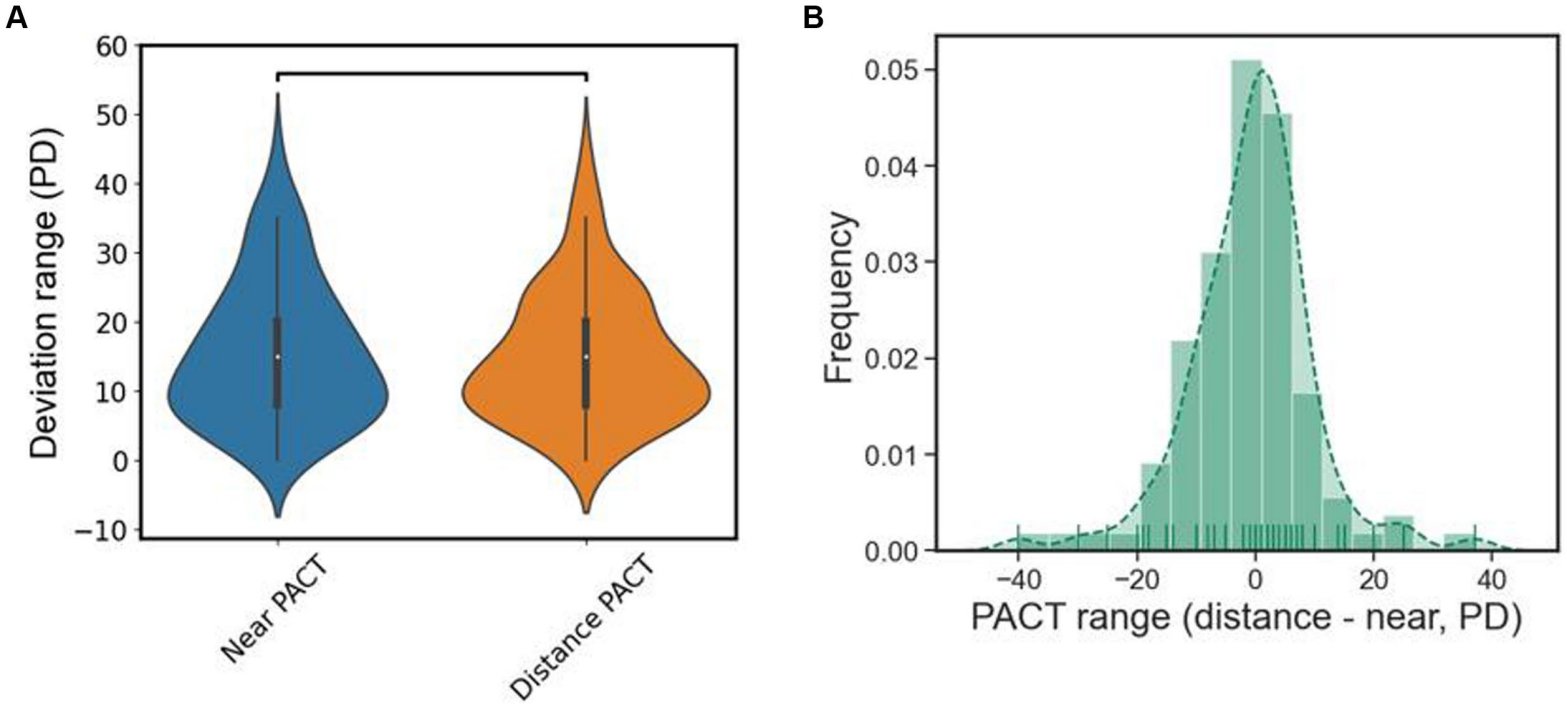
**(A)** The violin plot of the deviation range of PACT, there is no statistical difference in distance and near. **(B)** Subtract the results of the PACT deviation range at distance from the PACT deviation range at near and plot it as a histogram, it roughly normally distributed.

Due to the phenomenon of “eating up prisms” in patients with type III acute acquired comitant esotropia (AACE), there is a certain range between the minimum and maximum values of PACT, which we refer to as the deviation angle range (PACT range). It reflects the ability of patients with type III AACE to conceal ocular deviation within a certain range. According to the results in Table 2, the deviation angle range is approximately the same in both far distance (15.11 PD) and near distance (15.72 PD) examinations, with an average of 15.42 PD. To further explore the differences in PACT range between far and near distances, we represented the results of PACT range for far and near distances in the form of a violin plot for easier observation. As shown in Figure 6A, there was no significant statistical difference between the PACT range at far distance and that at near distance. We calculated the difference between the PACT range for each patient at far and near distances and plotted it using a histogram. And as shown in Figure 6B, the distributions of both were approximately centered around 0 PD, indicating a normal distribution, further illustrating that the numerical values of PACT range are roughly equal between far and near distances.

### 3.4 Postoperative effect and analysis

**Figure 7:**
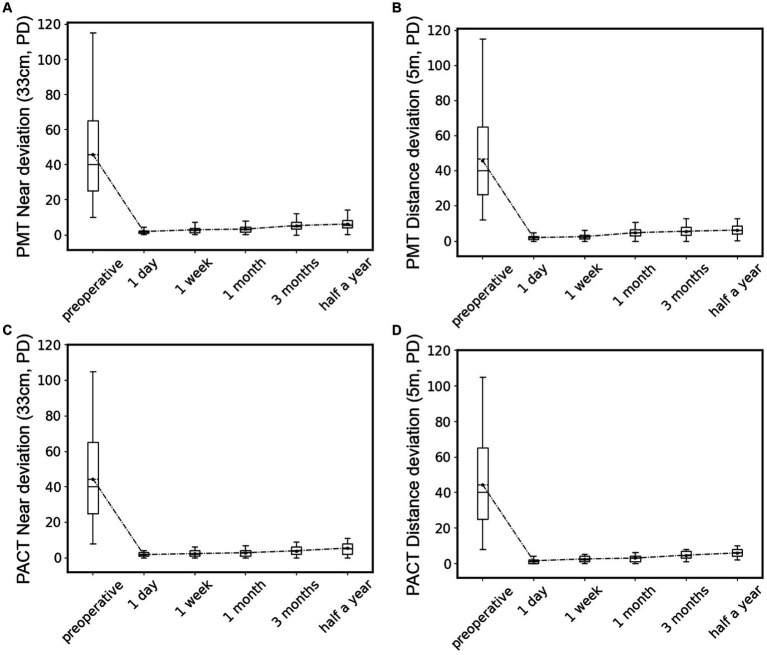
Statistical box plots of patients’ deviation angle preoperative and 1 day, 1 week, 1 month, 3 months, and 6 months after surgery, test by PMT at near location **(A)** and distant location **(B)**, or by PACT at near location **(C)** and distant location **(D)**.

We further analyzed the postoperative outcomes of patients with type III acute acquired comitant esotropia (AACE). As shown in Figure 7, we conducted deviation angle examinations using both PMT and PACT methods before surgery and at postoperative intervals of 1 day, 1 week, 1 month, 3 months, and 6 months, and performed statistical analysis. We found that after surgical treatment, the magnitude and variability of esotropia significantly decreased in patients, with the alternating cover test showing no movement or esotropia at postoperative day 1. The esotropia values were lowest at postoperative day 1 and showed a gradual increase over time, with a similar pattern observed in both near and far distance examinations. However, none of the patients reported a recurrence of diplopia symptoms, indicating successful treatment outcomes for all type III patients without recurrence.

## 4 Discussion

In recent years, the prevalence of type III AACE has significantly increased, particularly with the growing duration of near-work activities in the population, especially excessive use of smartphones. Our previous research also identified that increased daily near-work time and uncorrected myopia during near-work are independent risk factor for the onset of type III AACE (20). In this study, we observed a similar pattern where, following the outbreak of the COVID-19 pandemic, there was an explosive growth in type III AACE cases, likely attributed to reduced outdoor activities and prolonged use of electronic devices. This increase may be associated with increased tension in the medial rectus muscles due to prolonged near work, convergence spasm induced by myopia, and compensatory esotropia (11, 12). We conducted a retrospective analysis of 108 cases of type III AACE, stratifying them into adult and pediatric groups for statistical observation. We found that the number of cases in the adult group surged 1–2 years later than in the pediatric group, and the duration of disease at the initial visit was significantly longer in the adult group compared to the pediatric group. We speculate that this difference may be due to adults having higher tolerance to abnormal accommodation and convergence compared to children, or it may be related to parents of pediatric patients being more concerned about the visual disturbances affecting their children’s learning and daily life, prompting earlier medical consultations. However, the etiology of AACE is not yet fully understood, and further research is needed to explore its mechanisms in the future.

Regarding the treatment of type III AACE, surgery is currently widely employed as the primary therapeutic approach. Due to the notable phenomenon of “prism adaptation” in AACE patients, relying solely on traditional PACT for assessing the deviation angle and using the prism diopter at which the eye movement disappears upon uncovering (referred to as PACTmin) as the target surgical dosage often leads to postoperative under-correction or long-term recurrence (21). Therefore, the ability to expose the maximum deviation angle preoperatively in type III AACE patients becomes a crucial factor in determining the success of surgery. Zhou et al. (16) proposed a method in which the preoperative PACT results (referred to as PACTmin in this paper) are used as the target surgical dosage, and intraoperatively, the surgery is adjusted incrementally based on whether the patient’s subjective diplopia symptoms disappear until diplopia is resolved. By retrospectively analyzing the surgical increments required by these AACE patients, they are used as a reference for the target surgical dosage in other AACE patients. However, we believe that the incremental data obtained from postoperative retrospective analysis of surgical dosage may not accurately reflect the true deviation angle in AACE patients, as we found that the same PACTmin may correspond to multiple different PMT values, making it challenging to generalize and apply clinically. Dai et al. (22) used the recovery point from PACT (referred to as PACTmax in this paper) as the target surgical dosage design and achieved significant and stable improvements postoperatively, indicating that using PACTmax as the target surgical dosage design for AACE can yield significant results.

PMT examination is only suitable for patients with mature binocular vision, which is exactly the case for type III AACE patients who are typically older children or adults with some level of binocular vision. This study found that PMT, as a subjective method for measuring deviation angle, showed no significant difference compared to the objective PACTmax. Moreover, since PMT examination can completely disrupt fusion, it suggests that PMT can expose the maximum deviation angle in type III AACE. Our research found that there was no significant statistical difference between PMT and PACTmax, whether in distance or near examination. Therefore, we propose using PMT as the target surgical dosage for the surgical treatment of type III AACE.

In this study, we also investigated the performance of PACT range in both distance and near examinations. Our research revealed that there was no significant statistical difference in PACT range between distance and near examinations, indicating a substantial “buffer zone” in PACT examinations for type III AACE patients. This buffer zone is the main cause of the “eating prism” phenomenon. Due to insufficient understanding of type III AACE among many ophthalmologists, PACTmin is often used as the target surgical amount, leading to postoperative under-correction or recurrence. Because PACT recovery values and PAT can expose the maximum deviation angle of type III AACE patients, some scholars in previous studies have also used these two examination methods as target surgical amounts, achieving good results. If referring to the PAT examination used in previous studies, regular replacement of prisms is required, which may take weeks or even longer, and during this period, frequent replacement of prism lenses is needed, incurring significant diagnostic and treatment costs. Therefore, using PMT as the target surgical amount can not only achieve good therapeutic effects but also effectively reduce the number of patient visits and the associated costs, making it worth promoting as a method for determining the target surgical amount for type III AACE patients in clinical practice. This study was retrospective, with complete follow-up data collected up to 6 months postoperatively, but lacked a comprehensive assessment of binocular visual function. No control group was set up in this study, and further support from large-sample clinical observations and prospective studies is needed to validate the research conclusions.

In conclusion, preoperative exposure of the maximum deviation angle serves as a crucial factor for the success of surgery in type III AACE. We believe that preoperative PMT examination can expose the maximum deviation angle in type III AACE, offering a rapid, effective, and cost-effective method. Utilizing PMT examination results as the target surgical amount can lead to favorable outcomes for type III AACE patients, including the elimination of diplopia and correction of esotropia. Therefore, it is worthy of clinical promotion and application.

## Data availability statement

The raw data supporting the conclusions of this article will be made available by the authors, without undue reservation.

## Ethics statement

The studies involving humans were approved by Branch for Medical Research and Clinical Technology Application, Ethics Committee of First Affiliated Hospital of Fujian Medical University. The studies were conducted in accordance with the local legislation and institutional requirements. Written informed consent for participation in this study was provided by the participants' legal guardians/next of kin.

## Author contributions

HW: Funding acquisition, Investigation, Software, Writing – original draft. WZ: Conceptualization, Data curation, Project administration, Resources, Supervision, Validation, Writing – review & editing.
